# Novel natural killer cell-based therapies for hematologic and solid malignancies: latest updates from ASCO 2024

**DOI:** 10.1186/s13045-024-01575-0

**Published:** 2024-07-29

**Authors:** Xubo Gong, Lianjun Zhang, Xin He, Jing Yang, Xiang Li, Weiwei Liu, Bin Zhang, Zhihua Tao, Wenbin Qian

**Affiliations:** 1 Department of Clinical Laboratory, The Second Affiliated Hospital, Zhejiang University School of Medicine, Hangzhou, Zhejiang 310000 P.R. China; 2 Department of Hematological Malignancies Translational Science, Gehr Family Center for Leukemia Research, Hematologic Malignancies and Stem Cell Transplantation Institute, Beckman Research Institute, City of Hope Medical Center, Duarte, CA USA; 3 Department of Hematology, The Second Affiliated Hospital, Zhejiang University School of Medicine, Hangzhou, 310000 P.R. China

**Keywords:** Natural killer cell, Solid tumor, Lymphoma, Relapsed or refractory tumor

## Abstract

Natural killer (NK) cell-based therapies have made great progress in treating both hematological and solid tumors. Their unique mechanism of action does not rely on antigen presentation to recognize and eliminate tumor cells, making them a promising approach for cancer immunotherapy. In this review, we present a comprehensive summary of the latest clinical data of the novel NK cell-based therapies from the 2024 American Society of Clinical Oncology (ASCO) Annual Meeting, highlighting the potential of these advancements to revolutionize the treatment of hematologic malignancies and solid tumors.

## To the editor

Unlike T cells, Natural killer (NK) cells could recognize and eliminate malignant cells independent of antigen presentation, making them particularly effective against various types of tumors [1]. This review summarizes the latest clinical findings of novel NK cell-based therapies as presented at the 2024 American Society of Clinical Oncology (ASCO) Annual Meeting.

### Adoptive transfer of modified NK cell

One Phase I/II study [2] evaluated the efficacy of modified NK cells, resistant to TGFβ-induced suppression and expanded from a universal donor pool, in combination with irinotecan, temozolomide, and dinutuximab in children with relapsed or refractory (R/R) neuroblastoma. After administration of up to six cycles of treatment, 75% of patients achieved a partial response (PR). It manifests that the treatment strategy is safe and feasible, with early objective responses noted in the preliminary patient cohort.

### NK cell engager

AFM24 is a bispecific, tetravalent innate cell engager binding CD16a on NK cells and epidermal growth factor receptor (EGFR) on solid tumors. AFM24 combined with atezolizumab was assessed in patients with EGFR-WT non-small cell lung cancer [3]. After a median duration of 14.4 weeks, the objective response rate (ORR) was 26.7%, and no unexpected toxicities were observed compared to each single agent. It demonstrates that this combination is well-tolerated and shows a manageable safety profile in these patients.

### Cytokine-based NK cell activation

The safety and tolerability of JCXH-211, a self-replicating mRNA encoding IL-12, were assessed in patients with malignant solid tumors in a phase I study [4]. Patients were administrated across three dose levels, and grade ≥ 3 treatment-related adverse events (TRAEs) occurred in 10% of patients. It reveals a favorable safety profile, and antitumor activities in the heavily pretreated late-stage patients.

Another phase I/IIa study [5] evaluated GI-102, a novel CD80-IgG4-IL2V3 fusion protein driving robust proliferation and activation of NK cells, in patients with advanced or metastatic solid tumors. The ORR was 17.4%, and grade ≥ 3 TRAEs were found in 15.6% of patients. It shows that GI-102 is well tolerated with meaningful monotherapy activity in patients who failed on standard of care.

LTC004, a novel IL-2Rβ/γ cytokine agonist designed to minimize toxicity with improved potency, was assessed in patients with advanced or metastatic solid tumors [6]. No dose-limiting toxicities was observed. The ORR was 5.9%, and the disease control rate was 58.8%. It demonstrates encouraging anti-tumor efficacy and is well tolerated in patients enrolled.

### NK cell activation by immune checkpoint inhibitor (ICI)

Lacutamab, an ICI targeting killer cell immunoglobulin-like receptor 3DL2 (KIR3DL2), was assessed in patients with R/R mycosis fungoides. With a median follow-up of 11.8 months, the ORR was 22.4%, and the median progression-free survival (PFS) was 10.2 months. Grade ≥ 3 TRAEs were observed in 3.7% of patients [7]. It substantiates the promising clinical efficacy of lacutamab, with a favorable safety and tolerability profile among these patients.

Elotuzumab is a signaling lymphocytic activation molecule family member 7 (SLAMF7) checkpoint inhibitor. A phase Ib/II trial [8] evaluated the combination of elotuzumab and belantamab mafodotin in patients with R/R myeloma. The PR was 40%, and grade ≥ 3 TRAEs occurred in 30% of patients. It shows an encouraging safety profile and a promising preliminary efficacy.

IMM0306, which activates NK cells via blockade of CD47-SIRPa interaction and FcɣR engagement, achieved an ORR of 30.3% in patients with R/R CD20-positive B-cell non-Hodgkin’s lymphoma [9]. The median PFS was 10.58 months, and grade ≥ 3 TRAEs occurred in 68.8% of patients. It indicates that IMM0306 is well-tolerated and has promising preliminary anti-tumor activity especially in patients with R/R follicular lymphoma and marginal zone lymphoma.

Of note, dual ICI with pembrolizumab (a PD-1 inhibitor) and favezelimab (a lymphocyte-activation gene 3 inhibitor) was evaluated in patients with anti-PD-1-naive R/R classical Hodgkin’s lymphoma [10]. With a median follow-up of 36.9 months, the ORR was 83%. The median PFS was 19.4 months, and 24-month overall survival rate was 93%. Grade ≥ 3 TRAEs occurred in 23% of patients. It demonstrates sustained antitumor activity and manageable safety in patients studied.

In conclusion, 2024 ASCO Annual Meeting underscored substantial advancements in NK cell-based therapies, including adoptive transfer of modified NK cells, NK cell engagers, cytokine- and ICI-based NK activation (Tables 1 and 2) strategies, highlighting their potential to revolutionize tumor treatment and offering optimism for patients with R/R tumors.

**Table 1:** Advancements of NK cell transfer, NK cell engagers and Cytokine-based NK cell activation for hematologic and solid tumors

Type of therapy	Adoptive NK cell transfer	NK cell engagers	Cytokine-based NK cell activation		
**Authors (reference)**	Ranalli [2]	Kim [3]	Le [4]	Lee [5]	Gong [6]
**Trial identifier (Phase)**	NCT04211675 (II)	NCT05109442 (I/II)	NCT05727839 (I)	NCT05824975 (I/II)	NCT05666635 (I)
**Agents**	UD-TGFβi NK cells + irinotecan + temozolomide + dinutuximab	AFM24 (a bispecific, tetravalent innate cell engager) + atezolizumab	JCXH-211, a self-replicating mRNA encoding IL-12	GI-102 (CD80-IL2v3)	LTC004, a novel IL-2Rβ/γ cytokine agonist
**Malignancy**	R/R Neuroblastoma	EGFR -WT non-small cell lung cancer	Malignant solid tumors	Advanced or metastatic solid tumors	Advanced solid tumors
**Patient number**	4	17	10	32	17
**Male**	N/A	82.4%	N/A	N/A	N/A
**Median Age and (or) range (years)**	7–11	66(45–75)	N/A	N/A	N/A
**Clinical trial design**	21-day cycles of CIT as per COG protocol ANBL1221 with UD-TGFbi NK cells (1 × 10^8^ cells/kg) infused on day 8	Phase 2 dose of 480 mg AFM24 was given IV weekly in combination with 840 mg atezolizumab IV fortnightly	JCXH-211 at 5, 25, 50, and 100 mg were given Q4W. Dose Limiting Toxicities were monitored for 28 days after first dose	GI-102 was administered IV Q3W until disease progression or unacceptable toxicities	Dose escalation part assessed safety and tolerability of LTC004 with doses ranging from 3.0 to 360 µg/kg IV Q3W
**Median Follow-up (months)**	N/A	5.5	N/A	N/A	N/A
**Response rate**	PR 75%	ORR 26.7%	NA	ORR 17.4%	ORR 5.9%, DCR 58.5%
**Survival**	N/A	N/A	N/A	N/A	N/A
**Summary**	It could be safely and feasibly administered to children with R/R NBL after treatment with CIT	It shows remarkable signs of clinical efficacy, even in patients with resistance to prior CPI, and a well-tolerated and manageable safety profile	It demonstrates good safety profile. Antitumor activities were observed in the heavily pretreated late-stage patients	GI-102 is well tolerated up to dose of 0.45 mg/kg Q3W, in patients who failed on standard of care	It shows encouraging anti-tumor efficacy, and well tolerated in patients with advanced or metastatic solid tumors

***CIT*** chemoimmunotherapy, ***CPI*** checkpoint inhibitor, ***DCR***, disease control rate, ***EGFR*** epidermal growth factor receptor, ***IV*** intravenously, ***LAG-3*** lymphocyte-activation gene 3, ***N/A*** not available, ***NBL*** Neuroblastoma, ***NHL*** non-Hodgkin lymphoma, ***NK*** Natural killer, ***NKG2A*** NK cell lectin-like receptor subfamily C member 1, ***ORR*** objective response rate, ***PR*** partial response, ***Q3W***, once every 3 weeks, ***R/R*** relapsed or refractory, ***TGF*β*i*** TGFβ-induced suppression, ***UD*** universal donor, ***WT*** wide type

**Table 2:** Advancements of NK cell activation immune checkpoint inhibitor for hematologic and solid tumors

Type of therapy	NK cell activation via immune checkpoint inhibitors			
**Authors (reference)**	Porcu [7]	Browning [8]	Yang [9]	Timmerman [10]
**Trial identifier (Phase)**	NCT03902184 (II)	NCT05002816 (I/II)	NCT05805943 (I)	NCT03598608 (I/II)
**Agents**	Lacutamab, an ICI of KIR3DL2	Elotuzumab(an ICI of SLAMF7) + belantamab mafodotin	IMM0306, a fusion protein of CD20 with the CD47 binding domain of SIRPa	Favezelimab (anti–LAG-3 monoclonal antibody) + pembrolizumab (a PD-1 inhibitor)
**Malignancy**	R/R mycosis fungoides	R/R MM	R/R CD20-positive B-cell non-Hodgkin’s lymphoma	R/R classical Hodgkin lymphoma
**Patient number**	107	12	48	30
**Male**	N/A	N/A	30%	N/A
**Median Age and (or) range (years)**	62	66.5 (59–79)	56	N/A
**Clinical trial design**	Lacutamab 750 mg was administered as an IV infusion until disease progression or unacceptable toxicity	Elotuzumab 10 mg/kg IV on days 1, 8, 15, 22 every 28 days for cycles 1 and 2; followed by 20 mg/kg on day 1 of each 28-day cycle. Belantamab mafodotin IV with the starting dose of 1.9 mg/kg IV at every 4-week interval	IMM0306 was administered at escalating doses of 0.04, 0.1, 0.25, 0.5, 0.8, 1.2, 1.6, 2.0 mg/kg IV once a week until disease progression or intolerable toxicity	Pembrolizumab 200 mg IV Q3W plus favezelimab 200 mg starting dose, then dose escalation to 800 mg IV Q3W; and a dose expansion phase (pembrolizumab 200 mg Q3W plus favezelimab 800 mg Q3W for up to 35 cycles)
**Median Follow-up (months)**	11.8	N/A	N/A	36.9
**Response rate**	ORR 22.4%	PR 40%	ORR 30.3%	ORR 83%
**Survival**	Median PFS 10.2 months	N/A	Median PFS 10.58 months	Median PFS 19.4 months, 24-month OS rate 93%
**Summary**	It confirms promising clinical activity of lacutamab, with a favorable safety and tolerability profile	The combination indicates an encouraging safety profile and a promising preliminary efficacy in those patients	IMM0306 is well-tolerated and with promising preliminary anti-tumor activity especially in patients with R/R FL and MZL	It demonstrates sustained antitumor activity and manageable safety in patients studied

***CPI*** checkpoint inhibitors, ***FL*** follicular lymphoma, ***IV*** intravenously, ***KIR3DL2*** Killer cell immunoglobulin like receptor 3DL2, ***MM*** multiple myeloma, ***MZL***: marginal zone lymphoma, ***N/A*** not available, ***NK*** Natural killer, ***ORR*** objective response rate, ***PFS***: progression-free survival, ***PR*** partial response, ***Q4W*** once every 4 weeks, ***Q3W*** once every 3 weeks, ***R/R*** relapsed or refractory

## Data Availability

No datasets were generated or analysed during the current study.

## Abbreviations

CAR: Chimeric antigen receptor
CIT: Chemoimmunotherapy
CR: Complete remission
EGFR: Epidermal growth factor receptor
FL: Follicular lymphoma
MZL: Marginal zone lymphoma
IV: Intravenously
ICI: Immune checkpoint inhibitor
KIR3DL2: Killer cell immunoglobulin like receptor 3DL2
LAG-3: Lymphocyte-activation gene 3
MLA: Mesonephric-like adenocarcinoma
NBL: Neuroblastoma
NHL: Non-Hodgkin lymphoma
NK: Natural killer
NKG2A: NK cell lectin-like receptor subfamily C member 1
ORR: Objective response rate
OS: Overall survival
PR: Partial response
PFS: Progression-free survival
Q3W: Once every 3 weeks
R/R: Relapsed or refractory
TROP2: Trophoblast cell-surface antigen 2
TGFbi: TGFb-induced suppression
UD: Universal donor
WT: Wide type
